# Community-based Suicide Interventions in Rural United States: A Scoping Review

**DOI:** 10.1007/s10597-025-01480-x

**Published:** 2025-07-23

**Authors:** Christopher Weatherly, Maryam Abdelghani, Genesis Rebeca Cook, Maryam Irfan, Judy Meirose, Eleni Gaveras, Nicholas Johnson, Kimberly B. Roth

**Affiliations:** 1 School of Social Work, University of Georgia, Athens, GA USA; 2 Mercer University School of Medicine, Savannah, GA USA; 3 Mercer University School of Medicine, Macon, GA USA; 4 San Diego State University, Calexico, CA USA

**Keywords:** Suicide prevention, Rural health disparities, Community-based programming, Evidence-based practices, Rural mental health

## Abstract

Rural communities in the United States (US) are disproportionately burdened with higher suicide rates than non-rural ones, facing structural and cultural barriers that make it less likely for suicidal individuals to obtain help. Community-based interventions have been called for to address the need for integrative approaches relevant to the rural landscape. To facilitate increased adaptation/implementation of rural suicide prevention programming, we conducted a scoping review of the literature to evaluate the state-of-the-science. Following PRISMA-ScR guidelines, we searched for and identified relevant peer-reviewed literature across four databases, using dual screening/extraction throughout. We extracted information on key article characteristics, program descriptions, and methodological approaches to identify trends, gaps, and emergent themes. 29 articles were included in this review. Studies predominantly focused on suicide prevention for youth, particularly amongst Indigenous communities, with papers also concentrating on medical settings and US veterans. While a wide range of intervention and evaluation approaches were employed, few studies measured clinical outcomes, utilized theoretical frameworks or official rural definitions, or used comprehensive prevention strategies. This review offers investigators a guide to the existing evidence base and growing patterns in the field of suicide prevention in rural US areas. Results highlight the dearth of literature on prevention programming in this much needed yet overlooked area of study. Future intervention research should consider using context-specific rural classifications, relevant clinical outcomes, and comprehensive theory-backed strategies embedded in their approach.

**Supplementary Information:**

The online version contains supplementary material available at 10.1007/s10597-025-01480-x.

## Introduction

Suicide represents a continual and increasing public health concern within the United States (US), remaining one of the leading causes of death for the past two decades (Garnett et al., 2022). Suicide exerts an extensive emotional and psychological cost on individuals, families, communities, and society as a whole. Additionally, suicide contains cascading and tangible economic, social, and healthcare-related impacts (Shepard et al., 2016; WHO, 2023). Current evidence shows residents of rural areas are at higher risk of suicidal behaviors (Casant & Helbich, 2022; Kegler et al., 2017; Lee et al., 2023; Lemke et al., 2022). Suicide rates in rural America have remained consistently higher, increasing 46% since 2000 compared to 27% for urban settings (CDC, 2023; Runkle et al., 2023).

When unpacking these discrepancies, there are multiple structural and cultural factors to consider that make rural areas distinctly unique from urban environments. Structural factors include limited access to trained mental health providers, a shrinking healthcare workforce, increasing economic disparities, and lack of transportation (Hirsch & Cukrowicz, 2014; Mohatt et al., 2021). Cultural factors include social norms that stigmatize mental illness and discourage help-seeking behavior, high rates of gun ownership and other access to lethal means, and increasing geographic and social isolation (Fontanella et al., 2015; Mohatt et al., 2021). Several subpopulations residing within rural settings face heightened suicide risks due to social, environmental, psychological, and socio-demographic factors such as age, geographic barriers, history of mental illness, gender, ethnicity, employment, and economic status (Casant & Helbich, 2022; Mohatt et al., 2021). Rural populations who are more vulnerable to suicidal behaviors or who face disproportionate constraints to preventive services include American Indians/Alaska Natives (AI/AN), farm workers, young men, veterans, and people who are lesbian, gay, bisexual, transgender, or queer/questioning (LGBTQ +) (Freire & Koifman, 2013; Paley, 2022; Perry et al., 2022; Steelesmith et al., 2019).

While the current evidence base for suicide prevention programs in the US is growing, there is a lack of focus and attention when considering their applicability in rural contexts (Barnhorst et al., 2021). Rural areas present unique challenges for suicide treatment and prevention efforts. Designing and testing interventions are made more complicated as homes and residences are often more spread out, access to intervention points such as primary and behavioral health care and emergency medical services are limited, as well as differing cultural norms surrounding mental health stigma and gun ownership. However, rural communities also possess notable strengths that can be leveraged in suicide prevention efforts, including strong social ties, a deep sense of interdependence, and resilience in the face of ongoing social and institutional changes that reshape rural life (Brown & Schafft, 2011; Meit & Knudson, 2020). These unique challenges and strengths necessitate specialized and context-specific intervention strategies (Michael & Ramtekkar, 2022). Comprehensive community-based approaches have therefore been called for to address the need for targeted and coordinated strategies relevant to the varied landscapes and populations comprised within rural communities (Chu et al., 2017; NAASP, 2017).

**Fig. 1:**
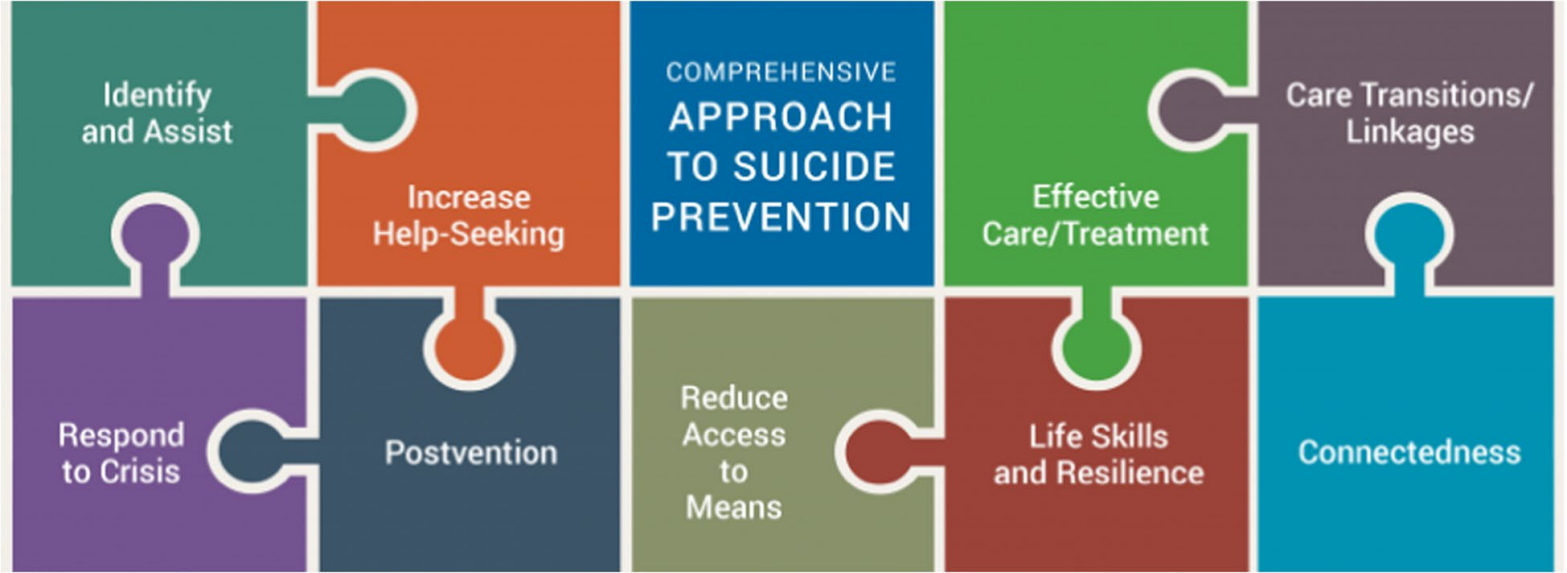
Elements of a comprehensive approach to suicide prevention. Note: taken from the Suicide Prevention Research Center’s website: https://sprc.org/effective-prevention/comprehensive-approach (accessed April 16, 2024)

Community-based interventions refer to mezzo-level approaches that center on defined groups within a specific community, including populations within hospitals, neighborhoods, schools, and workplace settings (Stone et al., 2017). A community-based suicide intervention approach is considered “comprehensive” when it addresses multiple ecological factors influencing suicidal behavior through theory-driven efforts that integrate prevention, treatment, and postvention strategies (APHA, 2021). The Suicide Prevention Resource Center (SPRC), funded by the US Department of Health and Human Services’ Substance Abuse and Mental Health Services Administration (SAMHSA), provides a framework for such a comprehensive approach (SPRC, 2020). This framework outlines nine strategies that can be advanced through an assortment of policies, programs, practices, and services. These include but aren’t limited to identifying and assisting persons at risk through screenings and gatekeeper training, as well as supporting safe care transitions and fostering organizational linkages through referral protocols and interagency agreements (see Fig. 1). By focusing on community settings instead of individual-led approaches, these strategies can offer additional reach for populations across the suicide risk spectrum and can complement other clinical interventions in multiple healthcare, education, or other social service environments (Stone & Crosby, 2014).

As US rural suicide rates continue to rise and multiple calls are put forward for community-based prevention efforts (Chu et al., 2017; NAASP, 2017) a current and comprehensive review and analysis of the existing literature is needed to identify patterns and gaps while highlighting future research needs and methodological considerations. Given the high priority of this public health issue, a systematic scoping review of the literature was conducted to synthesize efforts in this area and to facilitate increased research, adaptation, and implementation of community-based suicide prevention programs in rural US areas.

## Methods

To identify patterns and gaps in the evidence base, this study aimed to answer the following questions: 1. What are the key characteristics of the included studies (e.g., studied region, study aims, how rurality is defined)? 2. Which methodological considerations were taken by study authors (e.g., study design, measured outcomes, theoretical framework)? and 3. What are the characteristics of the implemented suicide prevention programs (e.g., target population, program description, strategies)? Given the broad scope of this paper's research objective, a scoping review was chosen as the methodological approach. Similar to systematic reviews, scoping reviews employ a rigorous, transparent, and exhaustive search strategy to identify and retrieve all relevant literature (Pham et al., 2014). However, systematic reviews aim to evaluate and synthesize relevant studies where study design and outcomes are often aligned. In contrast, scoping reviews are broader in scope and are used to provide an overview of a potentially diverse body of literature to find patterns, identify gaps, and map areas of future research and inquiry (Arksey & O’Malley, 2005).

This scoping review was conducted and reported according to the Preferred Reporting Items for Systematic Reviews and Meta-Analyses extension for Scoping Reviews (PRISMA-ScR) checklist (Tricco et al., 2018). A preliminary search of MEDLINE, the Cochrane Database of Systematic Reviews, and JBI Evidence Synthesis was conducted in April 2022 and determined that no systematic or scoping reviews on rural suicide interventions conducted within the US existed or were underway. The majority of existing reviews on suicide in rural areas examined either risk and protective factors (Freire & Koifman, 2013; Hirsch, 2006; Mohatt et al., 2021; Runkle et al., 2023) or compared the prevalence of suicide or suicide risks by level of urbanization (Barry et al., 2020; Casant & Helbich, 2022; Cox et al., 2013; Handley et al., 2011). One published scoping review on community-based suicide interventions in rural settings was identified; however, it focused only on adult populations in Australia (Dabkowski et al., 2022). As far as we know, this is the first scoping review to examine the width and breadth of all community-based suicide interventions in rural settings within the US.

### Search Strategy

The search strategy aimed to be systematic in scope and was developed in collaboration with a university librarian who assisted in creating the search strategies (see Appendix A for the full list of search terms). The librarian also aligned the search strategy in accordance to the JBI methodology for scoping reviews, which offers additional methodological guidance and is aligned with the PRISMA-ScR guidelines to ensure consistent reporting (Peters et al., 2020). After the initial limited search for articles on rural suicide prevention, key words were identified from titles and abstracts of selected articles and similar reviews to develop a comprehensive list of search terms. Three search strings were used for this review (“Suicide” AND “Intervention” AND “Rural”). Boolean logic and proximity operators were used to combine and refine the search terms contained within these search strings (see Appendix A for full list of search terms and number of results from each database).

The search strategy included four databases: EBSCO Medline, EBSCO PsycInfo, Scopus, and EBSCO CINAHL. After the completion of the title/abstract and full-text screening process (see below), the reference lists of all included articles as well as the previously identified reviews (Barnhorst et al., 2021; Barry et al., 2020; Casant & Helbich, 2022; Clifford et al., 2013; Cox et al., 2013; Dabkowski et al., 2022; Freire & Koifman, 2013; Handley et al., 2011; Hirsch, 2006; Hirsch & Cukrowicz, 2014; Mohatt et al., 2021; Runkle et al., 2023) were then hand searched to further ensure relevant studies were not overlooked. The initial database search for empirical articles took place in August 2022, with hand searching of references taking place in August 2023. Search limitations on language (English), peer-review status, and date were employed.

### Study Selection

The authors screened articles published in English from January 2000 through August 2022 to survey the scope of the literature over the past two decades. The study selection process included two phases: an initial title/abstract phase and a subsequent full-text screening process. In the title/abstract screening, a study was deemed eligible for full-text review if it (a) focused on the topic of suicide, including suicidal ideations, attempts, and deaths; (b) explicitly mentioned a rural setting in its description; (c) focused on an intervention through original research; and (d) was peer-reviewed (letters to the editor, commentaries, protocols, dissertations, and editorials were excluded).

Inclusion/exclusion criteria were developed by the first and last authors. For full-text screening, a study was deemed suitable for inclusion if it (e) was an evaluation of a community-level intervention with a primary aim of reducing suicide and/or suicide-related behaviors and (f) had an explicit focus on populations in a rural setting in the US. Following the CDC framework for community-based suicide prevention (Stone et al., 2017), community-based interventions were defined as mezzo-level approaches targeting defined groups or populations within a community setting. Studies were excluded if the suicide intervention strategy was micro- (e.g., pharmacologic treatments or individual psychotherapy) or macro-based (e.g., evaluating the effects of legislation related to dispensing drugs used for self-poisoning). For multisite studies, the study was only eligible for inclusion if results for rural areas were reported separately from non-rural ones. As the focus of this paper is on original research in rural communities, national-based studies referring to general populations were excluded, as were epidemiological studies and secondary data analyses.

Dual screening was used in both the title/abstract and full-text screening phases. For articles where decisions differed, a third screener (first or last author) was involved in discussions towards reaching group consensus. In the second screening phase, the first or last author was involved in all article screenings. The research team met regularly to resolve issues and share the decision-making process with the rest of the team to ensure continued understanding of the criteria. To streamline the study selection process, Covidence™, a systematic review management software, was employed to facilitate efficient collaboration among the research team and ensure a standardized and transparent approach to study selection (Harrison et al., 2020).

### Data Extraction

A dual extraction process was employed for the final identified articles. Covidence™ was again used to facilitate collaboration and transparency among the research team. As in the previous stage, all dual extractions were completed by either the first or last author. All disagreements were discussed by the first and last authors to reach consensus. To understand the full breadth and scope of the literature, the extraction template focused on study characteristics (i.e., location, study aims/conclusions), intervention descriptions, and methodological details. The authors also listed intervention strategies for each study based on the SPRC’s model on comprehensive approaches (SPRC, 2020). This classification method has been used in similar reviews and studies outlining gaps in rural suicide prevention programming (Bowersox et al., 2021; Roth et al., 2023).

### Collating, Summarizing, and Reporting the Results

Findings were summarized according to the research questions guiding this scoping review. Using constant comparison and consensus-building discussions (Mak & Thomas, 2022), the first and last authors assessed the extracted data and identified emerging themes and prevalent trends found in the reviewed articles.

## Results

**Fig. 2:**
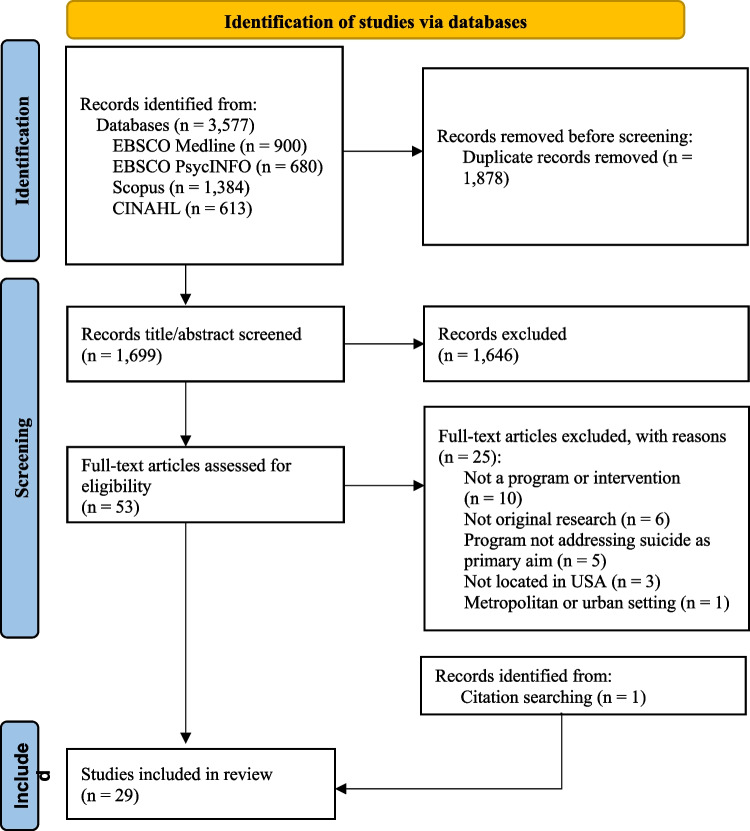
PRISMA diagram of study selection process. Flow chart of the scoping review From: Page MJ, McKenzie JE, Bossuyt PM, Boutron I, Hoffmann TC, Mulrow CD, et al. The PRISMA 2020 statement: an updated guideline for reporting systematic reviews. BMJ 2021;372:n71. https://doi.org/10.1136/bmj.n71

After the removal of duplicates, the database search yielded 1699 unique articles. Title and abstract screening then identified 53 articles for full-text review, after which 28 met the inclusion criteria. After hand searching both the included articles and related reviews, an additional study was identified. This resulted in a total of 29 included articles focusing on community-based suicide reduction efforts in rural US areas (see Fig. 2).

### Study Characteristics

Table 1 lists general characteristics of each article, including study aims, conclusions, descriptions of included prevention programs, and whether rurality was defined. Studies spanned thirteen years (2009–2022) with close to a third taking place in Alaska (n = 9, 31.0%) (Allen et al., 2009, 2018; Barnett et al., 2020; Mohatt et al., 2014; Trout et al., 2018; Wexler et al., 2019; Wexler et al. 2017a, b; White et al., 2022). Six studies (20.7%) were based in the South (Capps et al., 2019; Christensen-LeCloux et al., 2021; LeCloux et al., 2020; LoParo et al., 2019; Robertson et al., 2021; Schmidt et al., 2015), four (13.8%) in the West (Bailey et al., 2022; Ewert, 2021; McFaul et al., 2014; Story et al., 2016), three (10.3%) in the Midwest (Kim et al., 2022; Kohlbeck et al., 2021; Walker et al., 2009), two (6.9%) in Hawaii (Antonio et al., 2020; Chung-Do et al., 2015), and one (3.4%) in the Mid-Atlantic (Brancu et al., 2020). Four studies (13.8%) took place in multiple regions (Gujral et al., 2022; Pisani et al., 2018; Riblet et al., 2022; Wyman et al., 2010).

**Table 1:** Article characteristics and implemented interventions

Author(s)	Year	Aim(s)	Study Location^1^	Rurality Def	Program Name	Program Description	SPRC Categories	Main Conclusions
Allen et al	2009	To explore changes at the community level from a cultural prevention program	Southwest Alaska	None^2^	Qungasvik: Elluam Tungiinun (Toward Wellness)	Targets community-identified suicide prevention goals. Activities are developed by the community and compiled in Qungasvik, a toolbox used in designing modules	Life Skills & Resilience; Connectedness	Study was feasible and community readiness/protective factors increased in response to intervention
Allen et al	2018	To describe and test effectiveness of the intervention, comparing impact of high versus low intervention intensity	Southwest Alaska	None^2^	Qungasvik	Intervention provides individual, family, or community-level modules that promote protective factors for suicide protection. Process is not prescriptive and allows for adaptation to local customs	Life Skills & Resilience; Connectedness	Significantly greater growth in protection from suicide. Insight provided on “culture as treatment” and drawing from the world of local practices and Indigenous knowledge
Antonio et al	2020	To evaluate the impacts of the HCCI program based on qualitative data from youth leaders and HCCI community coordinators	Hawaii	None	The Hawai ‘i’s Caring Communities Initiative (HCCI) for Youth Suicide Prevention	Culturally tailored program that engages youth leaders, community members, and health professionals. Involves gatekeeper training, decreasing stigma, and increasing community suicide prevention knowledge and awareness	Identify & Assist; Increase Help-seeking; Life Skills & Resilience; Connectedness	Multiple multi-level impacts, including benefits and challenges of HCCI. Results also highlight the importance of involving youth as leaders in suicide prevention efforts
Bailey et al	2022	To assess the feasibility, acceptability, and fidelity of using extension agents to deliver intervention to rural students	Montana; Schools	USDA Economic Research Service (ERS) (by county)	Youth Aware of Mental Health (YAM)	Administered by adult instructors. Raises mental health awareness about risk and protective factors associated with suicide. Also enhances coping skills and emotional resiliency	Life Skills & Resilience	Feasible and acceptable for rural school settings. Provides support for using extension agents to teach mental health programs
Barnett et al	2020	To conduct a pilot evaluation of a 5-day culture camp developed in two remote regions of Alaska	Northwest Alaska; Culture Camps	None^2^	Youth Culture Camp	Camp participants participate in cultural activities, develop relationships with other youth plus elders and adult role models, and talk about their struggles	Life Skills & Resilience; Connectedness	All participants reported increased positive mood, sense of belongingness, and perceived internal ability to handle potential life stressors
Brancu et al	2020	To describe the feasibility of and adaptations made to the HOME program when implemented with rural patients	Mid-Atlantic; VA medical center	Rural–Urban Commuting Area Codes (RUCA): 2–10	Home-Based Mental Health Evaluation (HOME)	Intervention addresses barriers to initiating and maintaining outpatient care engagement, assesses suicide risk, and builds crisis management skills	Identify & Assist; Increase Help-seeking; Care Transitions/Linkages	Program is feasible to implement. Methods of addressing barriers to treatment engagement are offered
Capps et al	2019	To describe strengths, limitations, and results of replicating the PEACE protocol; to understand students’ access to lethal means when at risk for suicide	Western North Carolina; High School	Rural–Urban Continuum Codes (RUCC): 7	PEACE Protocol (The Prevention of Escalating Adolescent Crisis Events)	Provides a thorough and systematic evaluation of suicidal, homicidal, and self-injury risk for clinicians. Guides decision-making to develop and execute a response plan based on risk severity	Identify & Assist; Care Transitions/Linkages; Respond to Crisis; Reduce Access to Means	PEACE protocol can function in multiple ways to prevent rural student suicide. Findings also emphasize the importance of assessing students’ access to means
Christensen-LeCloux et al	2021	To evaluate the acceptability of universal suicide risk screening and preferred methods of implementation	Southern West Virginia	None	None	Patients fill out the suicide screening tool on a tablet. If screened positive, a provider conducts a brief follow-up, then provides disposition planning as needed	Identify & Assist	Universal suicide risk screening is valued and well-received by adult patients in rural primary care
Chung-Do et al	2014	To describe the youth leadership model utilized and provide reflections on important factors for success in implementing the youth suicide prevention project	Hawaiian Youth and community orgs	None	Mobilizing Communities At-Risk (MCAR): A Youth and Community Project	Initiative trains youth leaders and community members in suicide prevention, so to develop community-based awareness activities	Identify & Assist; Increase Help-seeking; Connectedness	Model demonstrates importance of prioritizing relationship-building and recognizing community expertise in program implementation. Factors to consider for similar interventions that engage youth to address health disparities are provided
Ewert	2021	To analyze community responses to a culturally grounded, men-centered suicide prevention campaign	Shasta County, California	None	Captain Awesome	The campaign uses posters, billboards, and social media to direct residents to a webpage listing mental health, suicide prevention, and anti-stigma resources	Increase Help-seeking	Male-targeted suicide prevention efforts have little salience in rural communities with high mental health stigma and limited treatment resources
Gujral et al	2022	To evaluate the association between distribution of video-enabled tablets and frequency of mental health service use, suicide-related behavior, and emergency department visits for rural veterans	Multiple Veterans Affairs (VAs) across the US	Rural–Urban Commuting Area Codes (RUCA): Other than 1 or 1.1	None	Patients receive video-enabled tablet to facilitate participation in home-based telehealth	None	Receipt of tablet was associated with increased use of mental health care via video, increased psychotherapy visits, and reduced suicide behavior and ED visits
Kim et al	2022	To analyze the impact of implementing EmPATH unit on hospital admission for emergency department patients presenting with suicidal behaviors	Midwestern academic medical center	None	EmPATH (The Emergency Psychiatric Assessment, Treatment, and Healing unit)	Patients are sent to a separate unit from emergency department, placed on suicidal precautions, and evaluated. Assessment and treatment of underlying factors to suicidal behavior is then initiated	Effective Care/Treatment; Care Transitions/Linkages; Respond to Crisis	Findings suggest EmPATH reduces hospitalizations and improves follow-up outpatient visits
Kohlbeck et al	2021	To investigate the utility of a systems-level, primary prevention suicide prevention strategy in reducing suicide in a rural Wisconsin county	Wisconsin	None	Three interventions: 1. Referral Mechanism; 2. Mindfulness; 3. Social Marketing Campaign	1. Frontline staff are trained to recognize warning signs and refer to screener, who connects youth to resources. 2. Students are educated on mindfulness and coping skills. 3. Mindfulness/anti-stigma messages and local behavioral resources are disseminated via website, social media, and in print	Identify & Assist; Increase Help-seeking; Effective Care/Treatment; Care Transitions/Linkages; Life Skills & Resilience; Connectedness	Results are preliminary but promising across all three programs. Having a strong implementation body and buy-in from key partners was essential in creating change at the systems level
LeCloux	2020	To evaluate the feasibility and impact of a suicide risk screening program in an adult, rural primary care practice	West Virginia; Adult primary care practice	None	Ask Suicide-Screening Questions (ASQ) Toolkit	A brief suicide safety assessment is provided by primary care providers. If needed, follow-up safety assessment and management are provided	Identify & Assist; Increase Help-seeking; Care Transitions/Linkages	Suicide risk screening program is feasible and acceptable to adults in rural primary care. Implementing ASQ Toolkit can improve suicide risk assessment and detection rates
LoParo	2019	To investigate the relationship between attending suicide prevention trainings on perceived confidence and implementation of best practices of suicide assessment/treatment	Georgia	None	None	Providers can attend a variety of trainings, including: Assessing and Managing Suicide Risk, Collaborative Assessment and Management of Suicidality, Dialectical Behavior Therapy, and Question Persuade Refer	Identify & Assist; Effective Care/Treatment	Findings show a small but significant benefit to multiple trainings for increasing confidence, and that behavioral health providers who are confident are more likely to incorporate best practices
McFaul et al	2014	To describe the evolution, development, and effectiveness of a training-based toolkit to improve suicide prevention knowledge and skills of primary care providers and other employees in health care settings	Colorado	None	Suicide Prevention Toolkit for Rural Primary Care	Toolkit includes best practices on suicide prevention for providers. Office protocols for various primary care disciplines are offered that detail specific actions to be taken when a suicidal patient is identified	Identify & Assist; Effective Care/Treatment; Care Transitions/Linkages	Providers had an improved sense of preparedness, knowledge of suicide, and confidence to assess and address suicide
Mohatt et al	2014	To determine if the proposed intervention is implementable in rural Alaska Native communities and if it produces measurable effects	Southwest Alaska	None^2^	Qungasvik: Elluam Tungiinun (Toward Wellness) & Yupiucimta Asvairtuumaller-kaa (Strengthening our Yup’ik Identity)	Both interventions used modules selected by the community from the Qungasvik toolbox. Modules address protective factors and are conceived as an individual, family, or community intervention	Life Skills & Resilience; Connectedness	Intervention was implementable within rural Yup’ik Alaska Native settings. Implementation in these settings is feasible when sufficient resources are available to sustain high levels of local commitment
Pisani et al	2018	To test the appeal and feasibility of Text4Strength and its potential to extend universal school-based suicide prevention	Unknown; High schools	None	Text4Strength (extension of Sources of Strength)	Uses variety of novel interactive text messaging sequences and leverages peer modeling and testimonials to strengthen protective factors for suicide prevention at point of high school entry	Increase Help-seeking; Life Skills & Resilience; Connectedness	The program was technically feasible, and students found texts to be fun, appealing, and easy to use. Most reported gaining awareness of their own feelings and learning new ways to cope
Riblet et al	2022	To describe: the degree to which local sites implemented the Brief Intervention and Contact (BIC) Program; barriers or facilitators; and overall effectiveness	US Dept. of Veterans Affairs (VA) medical centers	Rural–Urban Commuting Area Codes (RUCA): > 29% of VA site patients require rural address	The World Health Organization Brief Intervention and Contact (BIC) Program	Patients receive a brief educational session on suicide prevention at discharge after inpatient hospitalization. Patients then have seven follow-up contacts over three months with interventionist	Care Transitions/Linkages; Life Skills & Resilience	The BIC program was successfully implemented with high participation. The program was appealing to patients and providers and helped to improve treatment engagement after discharge
Robertson et al	2021	To evaluate the effectiveness of Mental Health First Aid in broadening the network of gatekeepers in rural communities	Mississippi	None	Mental Health First Aid (MHFA)	Eight hour curriculum that teaches individuals how to better understand, identify, and respond to signs of mental health disorders and crises, such as suicidal thoughts and behaviors, depression, anxiety, psychosis, and substance use disorders	Identify & Assist; Respond to Crisis	MHFA is perceived as effective and valuable and appears to increase the confidence and competence of Extension agents as community gatekeepers
Schmidt et al	2015	To document a rural school district’s effort to initiate, implement, integrate, and evaluate a suicide prevention program integrated into the district’s existing school mental health system	Maryland; School district	None	Yellow Ribbon Ask 4 Help (part of the Yellow Ribbon Suicide Prevention Program (YRSPP))	Two trainings are implemented by school mental health staff: Ask 4 Help! Suicide Prevention for Youth; and Be a Link! Suicide Prevention Gatekeeper Training. Both teach students to increase help-seeking behaviors for themselves or for others	Identify & Assist; Increase Help-seeking	Integration of program into school mental health programming was feasible. Students’ knowledge and behavior related to help-seeking on behalf of peers increased. The district was also able to identify and connect youths having suicidal thoughts to services
Story et al	2016	To evaluate Better Todays and examine how community health education was used to increase mental health literacy in rural communities in a way that respects local customs and cultural norms	Idaho	None	Better Todays/Better Tomorrows for Children’s Mental Health/Youth Suicide Prevention training (Better Todays)	Adult gatekeepers are trained on identifying signs and symptoms of mental disorders in children and youth as well as on how to detect and respond to suicide risk	Identify & Assist; Increase Help-seeking	Participants reported improved mental health literacy. Data suggest that trainings and customized program content may be an effective way to improve mental health literacy in rural communities
Trout et al	2018	To describe theoretical underpinnings, preliminary acceptability, and feasibility outcomes of a community health education and mobilization model for Indigenous youth suicide prevention	Northwest Alaska; Villages	None^2^	Promoting Community Conversations About Research to End Suicide (PC CARES)	This study focused on the first of nine learning circles (“Where We’ve Been and Where We Are Going"). Discussions are held after watching a film linking the youth suicide epidemic to breakdowns of intergenerational mentorship/support associated with colonialism	Connectedness	Results demonstrate that the model is feasible and acceptable as a framework for incorporating both research evidence and Indigenous ways of knowing. PC CARES builds capacity, confidence, and collective commitment to suicide prevention practice
Walker et al	2009	To provide evaluative data for the LifeSavers'peer support, suicide, and crisis prevention program	Southern Illinois	None^2^	LifeSavers	Three-day training"retreat"teaches students how to work as a team, listen without judgment, recognize signs of depression/suicidal thoughts, and enlist the aid of a counseling resource or other adult	Identify & Assist	Significant increase in knowledge and positive attitudes toward suicide prevention and self-esteem
Wexler et al	2017	To describe process and evaluation findings, make improvement recommendations, and discuss implications	Alaska	None^2^	The Youth Leaders Program (YLP)	Youth leaders and teacher program advisors are trained to recognize and promote protective factors and to address and reduce risks associated with drug/alcohol abuse, violence, and bullying	Life Skills & Resilience; Connectedness	Positive impacts on both youth leaders and other students. YLP appears to have a range of positive outcomes associated with reduced suicide risk
Wexler et al	2017	To describe theoretical and practical considerations, and assess preliminary feasibility, learning, and behavioral outcomes of a grassroots training-of-trainers model	Northwest Alaska; Villages	None^2^	Promoting Community Conversations About Research to End Suicide (PC CARES)	Facilitators host learning circles where community members and service providers discuss prevention strategies and best practices to prevent youth suicide and promote health. Curriculum is developed with community leaders and tailored for the cultural context	Identify & Assist	Facilitator reflections were positive overall, suggesting PC CARES is feasible, acceptable, and potentially impactful as a way to translate research to practice in under-resourced, rural Alaska Native communities
Wexler et al	2019	To assess process and preliminary learning and behavioral outcomes of PC CARES	Alaska	None	Promoting Community Conversations About Research to End Suicide (PC CARES)	Facilitators host learning circles where community members and service providers discuss prevention strategies and best practices to prevent youth suicide and promote health. Curriculum is developed with community leaders and tailored for the cultural context	Identify & Assist	Results indicate increased gains in self-perceived knowledge, skills, and relationships associated with prevention. Also, statistically significant increases in prevention activities of non-participants who were close to participants
White et al	2022	To examine the utilization of research evidence and its impact on subsequent actions among participants in PC CARES	Northwest Alaska; Villages	None	Promoting Community Conversations About Research to End Suicide (PC CARES)	Facilitators lead discussions with the community on suicide prevention research in nine “Learning Circles”. Curriculum is developed in collaboration with community leaders and tailored for the cultural context	Identify & Assist	Significant correlations with utilization of research evidence and participants'intent to use evidence. Navigating discordant information, centering relationships, and Indigenous worldviews were key
Wyman et al	2010	To examine the effectiveness of intervention in enhancing protective factors among peer leaders and high school students	New York and North Dakota; Schools	None	Sources of Strength	Youth leaders are trained to change peer norms and behaviors by encouraging communication with trusted adults for suicidal friends and identifying and using interpersonal and formal coping resources	Life Skills & Resilience; Connectedness	Training of peer leaders with the Sources of Strength curriculum led to changes in norms across the full population of high school students

^1^Study setting is provided after the semicolon wherever applicable

^2^No official measurement offered, but rural characteristics of the community are described in detail

While all included studies explicitly labeled their studied region as “rural”, the majority (n = 24, 82.8%) did not report how rurality was determined. Only five studies (17.2%) outlined how rurality was operationalized, with four using Rural–Urban Commuting Area (RUCA) Codes (Brancu et al., 2020; Capps et al., 2019; Gujral et al., 2022; Riblet et al., 2022) and one using rural classifications designated by the US Department of Agriculture Economic Research Service (USDA ERS) (Bailey et al., 2022).

More than half (n = 19, 65.5%) of the included studies focused on youth populations. Eleven of these (37.9% of overall papers) studied suicide programs developed for Indigenous youth in either Alaska or Hawaii (Allen et al., 2009, 2018; Antonio et al., 2020; Barnett et al., 2020; Chung-Do et al., 2015; Mohatt et al., 2014; Trout et al., 2018; Wexler et al., 2019; Wexler et al., 2017a, b; White et al., 2022). The remaining eight (27.6%) targeted adolescents or young adults in other rural contexts (Bailey et al., 2022; Capps et al., 2019; Kohlbeck et al., 2021; Pisani et al., 2018; Schmidt et al., 2015; Story et al., 2016; Walker et al., 2009; Wyman et al., 2010). Five studies (17.2%) focused on patients in medical settings (Christensen-LeCloux et al., 2021; Kim et al., 2022; LeCloux et al., 2020; LoParo et al., 2019; McFaul et al., 2014), while three (10.3%) examined suicide prevention efforts for US military veterans within the Veterans Affairs (VA) healthcare system (Brancu et al., 2020; Gujral et al., 2022; Riblet et al., 2022). For the remaining two papers, one was developed for multiple populations in a rural and agricultural setting (Robertson et al., 2021), with the other targeting adult males (Ewert, 2021).

### Methodological Approaches

Table 2 presents a comprehensive summary of methodological considerations for each study organized by targeted population. All included studies represent a variety of theoretical, strategic, and methodological approaches in measuring program impacts. Close to half used a mixed-method design (n = 14, 48.3%), followed by strictly quantitative (n = 12, 41.4%) and qualitative (n = 3, 10.3%) approaches. Most (n = 22, 75.9%) did not report a theoretical framework guiding intervention design. Of the seven studies that did employ a framework, all but one focused on Indigenous youth in Alaska (Allen et al., 2009, 2018; Barnett et al., 2020; Trout et al., 2018; Wexler et al., 2017a; White et al., 2022). The remaining article focusing on high school students in the states of New York and North Dakota (Wyman et al., 2010). For the studies focusing on Indigenous youth in Alaska, there was predominate focus on decolonial and culturally-bound frameworks.

**Table 2:** Overview of methodological considerations of included studies organized by targeted population for intervention

	Author(s)	Year	Program Name	Study Population	Methodology	Trial Type	Outcome Type	Outcome	Theory of Suicide
								Clinical Outcome? (y/n)	
Indigenous youth	Antonio et al	2020	Hawai ‘i’s Caring Communities Initiative (HCCI) for Youth Suicide Prevention	Adult intervention coordinators; Youth intervention leaders (Ages 13–18)	Qualitative	Formative/ Implementation	Effectiveness	Multiple program themes, each align at an individual, interpersonal, community, or sustainability level	None
								N	
	Chung-Do et al	2014	Mobilizing Communities At-Risk (MCAR): A Youth and Community Project	Indigenous youth; Community intervention leaders; Youth intervention leaders	Quantitative/ Qualitative	Quasi-experimental	Process; Effectiveness	Number of people trained/ reached/ identified as at-risk for suicide; Number of trainings conducted	None
								N	
	Trout et al	2018	Promoting Community Conversations About Research to End Suicide (PC CARES)	Intervention facilitators and adult community members	Qualitative	Formative/ Implementation	Implementation	Feasibility; Acceptability	Decolonial framework
								N	
	Wexler et al	2017	Promoting Community Conversation About Research to End Suicide (PC CARES)	Intervention facilitators	Quantitative/ Qualitative	Quasi-experimental pilot study	Effectiveness	Facilitator readiness/ satisfaction; Participant understanding of content; Fidelity; Accuracy	None
								N	
	Wexler et al	2019	Promoting Community Conversations About Research to End Suicide (PC CARES)	Community residents or service providers, age 15 or older	Quantitative/ Qualitative	Quasi-experimental pilot study	Process; Implementation; Effectiveness	Fidelity; Accuracy; Perceived knowledge; Attitudes on prevention/ intervention; Sense of community of practice; Participation; Prevention behaviors; Social impact	None
								N	
	White et al	2022	Promoting Community Conversation About Research to End Suicide (PC CARES)	Intervention facilitators and adult community members	Quantitative/ Qualitative	Formative/ Implementation	Implementation	Utilization of research evidence factors; Participant interpretations of curriculum; Intentions to act on suicide prevention information; Fidelity; Accuracy	Decolonial framework
								N	
	Allen et al	2009	Qungasvik: Elluam Tungiinun (Toward Wellness)	Indigenous youth (Ages 12–17); Parents or adult sponsors; Adult key informants	Quantitative/ Qualitative	Quasi-experimental	Effectiveness	Community readiness; Community protective factors	Qasgiq (place of learning); Ellangneq (always being aware)
								N	
	Allen et al	2018	Qungasvik	Indigenous youth (Ages 12–17)	Quantitative	Quasi-experimental	Effectiveness	Reflection on consequences from alcohol; Reasons for living	Yup’ik Indigenous theory of change
								N	
	Mohatt et al	2014	Qungasvik: Elluam Tungiinun (Toward Wellness) & Yupiucimta Asvairtuumallerkaa (Strengthening our Yup’ik Identity)	Indigenous youth (7th-12th grade)	Quantitative	Formative/ Implementation	Implementation	Adherence; Quality; Dosage; Reach; Feasibility	None
								N	
	Barnett et al	2020	Youth Culture Camp	Indigenous youth (Ages 13–18)	Quantitative	Quasi-experimental pilot study	Effectiveness	Affect; Mastery; Interpersonal needs; Mattering; Self-esteem	Interpersonal theory of suicide
								Y	
	Wexler et al	2017	Youth Leaders Program (YLP)	Youth intervention leaders (Grades 8–11); Youth intervention advisors (teachers & village adults); Principals; Non YLP students (Grades 3–12)	Quantitative/ Qualitative	Quasi-experimental study	Effectiveness	Mattering; Affect; Substance use; Perceptions of effect on youth suicide	The transactional–ecological framework
								Y	
Children, adolescents, or young adults	Story et al	2016	Better Todays/ Better Tomorrows Youth Suicide Prevention Program	Parents and adult gatekeepers (School/ justice system personnel, police, volunteers, clergy, mayors, tribal social workers)	Quantitative	Quasi-experimental	Effectiveness	Mental health literacy	None
								N	
	Walker et al	2009	LifeSavers	High school students	Quantitative	Quasi-experimental	Effectiveness	Self-esteem; Self-acceptance; Knowledge/ attitudes toward helping	None
								Y	
	Capps et al	2019	PEACE Protocol (The Prevention of Escalating Adolescent Crisis Events)	High school students	Quantitative	Formative/ Implementation	Process	Number/ percentage of crisis codes; Characteristics and sequelae of crisis events (e.g., plan, referrals, hospitalizations)	None
								Y	
	Wyman et al	2010	Sources of Strength	High school students; Peer leaders	Quantitative/ Qualitative	Group-randomized trial	Effectiveness	Suicidal Ideation; Suicide perceptions and norms; Social connectedness; Peer leader behaviors	1) Diffusion of innovations theory 2) Valente’s social network thresholds model
								Y	
	Pisani et al	2018	Text4Strength (extension of Sources of Strength)	9th grade students	Quantitative/ Qualitative	Quasi-experimental study	Process; Implementation	Interaction with texts/ videos; Appeal; Perceived benefit	None
								Y	
	Kohlbeck et al	2021	Unnamed three intervention combination: Referral mechanism; Mindfulness; Social marketing campaign	Youth; Young adults; Frontline staff (i.e., teachers)	Quantitative/ Qualitative	Formative/ Implementation	Process; Implementation; Effectiveness	Reach; Efficacy; Adoption; Maintenance; Mindfulness-based stress reduction; Social media visits; Multiple community mental health outcomes	None
								Y	
	Schmidt et al	2015	Yellow Ribbon Ask 4 Help (part of the Yellow Ribbon Suicide Prevention Program (YRSPP))	Middle & High school students	Quantitative	Quasi-experimental study	Effectiveness	Suicidal risk/ ideation; Student knowledge related to suicide, help seeking, and aspects of program	None
								Y	
	Bailey et al	2022	Youth Aware of Mental Health (YAM)	High school students	Quantitative/ Qualitative	Formative/ Implementation	Implementation	Feasibility; Acceptability; Fidelity; Extension agent experiences	None
								N	
Medical Patients	LeCloux	2020	Ask Suicide-Screening Questions (ASQ) Toolkit	Adult primary care patients	Quantitative	Quasi-experimental pilot study	Implementation; Effectiveness	Feasibility; Proportion of patients screened; Suicide risk detection rates	None
								N	
	Kim et al	2022	EmPATH (The Emergency Psychiatric Assessment, Treatment, and Healing unit)	Adult emergency department patients with suicidal ideation or suicidal attempt	Quantitative	Quasi-experimental	Effectiveness	Inpatient psychiatric admissions	None
								Y	
	McFaul et al	2014	Suicide Prevention Toolkit for Rural Primary Care	Primary care providers; Clinical staff	Quantitative/ Qualitative	Quasi-experimental pilot study	Implementation; Effectiveness	Preparedness to screen; Knowledge of suicidal behavior; Opinions on working with suicidal patients	None
								N	
	Christensen-LeCloux et al	2021	Unnamed primary care suicide risk screening intervention	Adult primary care patients	Quantitative/ Qualitative	Quasi-experimental pilot study	Implementation	Previous screening experience; Acceptability; Subjective reactions/ preferences to screening experience	None
								N	
	LoParo	2019	Unnamed suicide prevention trainings intervention	Community behavioral health providers	Quantitative	Quasi-experimental	Effectiveness	Knowledge about suicide prevalence; Beliefs about suicide; Current practices to address suicide; Skill confidence	None
								N	
Veterans	Brancu et al	2020	Home-Based Mental Health Evaluation (HOME)	Veterans discharged from inpatient psychiatric unit	Quantitative	Formative/ Implementation	Process; Implementation	Phone calls; Home visits; Treatment engagement	None
								N	
	Gujral et al	2022	Unnamed video enabled tablet intervention	Veterans Affairs (VA) mental health care patients; Veterans considered high-risk for suicide by VA	Quantitative	Quasi-experimental study	Effectiveness	Psychotherapy/ medication management visits; Suicide risk; Likelihood of emergency department visit; Suicide behavior; Overdose reports	None
								Y	
	Riblet et al	2022	World Health Organization Brief Intervention and Contact (BIC) Program	Veterans discharged from inpatient psychiatric unit; Veterans Affairs (VA) site mental health providers	Quantitative/ Qualitative	Formative/ Implementation	Implementation	Multiple measures used to assess each domain of RE-AIM framework; Patient/ team/ staff satisfaction	None
								N	
Other	Ewert	2021	Captain Awesome	Adult wildfire survivors; Mental health professionals	Qualitative	Qualitative evaluation	Other: Qualitative themes	Multiple community responses: Stigma for men seeking help; Women and stigma; Beliefs about symptom severity and help-seeking; Material realities of rural life	None
								N	
	Robertson et al	2021	Mental Health First Aid (MHFA)	Mississippi Extension agents	Quantitative/ Qualitative	Quasi-experimental	Implementation; Effectiveness	Intervention skill use; Confidence in skill use; Barriers	None
								N	

While many studies used effectiveness evaluation measures within their assessment of program impacts (n = 19, 65.5%), close to a third (n = 9, 31.0%) were designed to only measure process and/or implementation outcomes. Only one of the effectiveness studies reported a randomized design (Wyman et al., 2010) with the rest employing quasi-experimental methods. Of the 19 effectiveness studies, nine reported a clinical outcome (Barnett et al., 2020; Capps et al., 2019; Gujral et al., 2022; Kim et al., 2022; Kohlbeck et al., 2021; Schmidt et al., 2015; Walker et al., 2009; Wexler et al., 2017a; Wyman et al., 2010), defined as measurable change in symptoms, health, functioning, quality of life, or survival (Ferreira & Patino, 2017). The ten remaining effectiveness studies measured factors such as mental health literacy (Story et al., 2016), participant understanding of intervention content (Wexler et al., 2017b; White et al., 2022), and broader social impacts (Wexler et al., 2019; Wyman et al., 2010).

### Intervention Characteristics

Table 1 includes brief descriptions of the implemented interventions in the included studies. Aside from four papers studying Promoting Community Conversations About Research to End Suicide (PC CARES) (Trout et al., 2018; Wexler et al., 2019; Wexler et al., 2017b; White et al., 2022) and three employing the Qungasvik (Allen et al., 2009, 2018; Mohatt et al., 2014) intervention approach, each remaining intervention in the included body of research was distinct. The PC CARES and Qungasvik interventions were carried out by two separate research groups, and each targeted Indigenous youth populations in Alaska. PC CARES is a multilevel and flexible model designed for Indigenous communities in Northwest Alaska. It employs community-led learning circles to cultivate learning on suicide prevention (Wexler et al., 2016). The Qungasvik intervention was designed for Yup’ik youth in Southwest Alaska and implements modules that promote protective factors. Similar to PC CARES, the intervention is not a prescriptive manual, but instead lays out a process for adapting each module’s activities to reflect local cultures, customs, and circumstances (Allen et al., 2009).

Six of the eight non-Indigenous studies targeting children, adolescents, or young adults focused on middle and high school students (Bailey et al., 2022; Capps et al., 2019; Pisani et al., 2018; Schmidt et al., 2015; Walker et al., 2009; Wyman et al., 2010). The remaining two articles focused on adolescence to age 24 (Kohlbeck et al., 2021; Story et al., 2016). Five studies were implemented within schools and used varied techniques such as employing a risk assessment tool (Capps et al., 2019), training peer leaders (Wyman et al., 2010), hosting student retreats with listening circles and activities (Walker et al., 2009), employing interactive text messages (Pisani et al., 2018), and implementing a multi-tiered systems-level strategy of referrals, mindfulness, and outreach (Schmidt et al., 2015). Three of the five studies housed in medical settings took place in adult primary care clinics (Christensen-LeCloux et al., 2021; LeCloux et al., 2020; McFaul et al., 2014), with single studies occurring in an emergency department (Kim et al., 2022) and community clinic setting (LoParo et al., 2019). All medical setting studies focused on varied approaches to building or improving suicide risk screening-based interventions. Of the three studies targeting veterans, two focused on discharge and transition processes from inpatient psychiatric to outpatient-level care (Brancu et al., 2020; Riblet et al., 2022). The third study connected at-risk VA patients with video-enabled telehealth tablets to address mental healthcare access barriers and needs (Gujral et al., 2022).

For the two remaining studies, one measured skills and confidence gained after a gatekeeper training for mental health first aid for agents in a Cooperative Extension office, which is an education-based and community outreach organization. Originally established to address rural and agricultural issues, Cooperative Extension has since expanded its programs to serve both rural and urban communities across the US (Robertson et al., 2021; USDA, 2025). The other paper examined how residents interpreted a website, social media, and billboard-based suicide prevention campaign for men (Ewert, 2021).

### Intervention Strategies

**Fig. 3:**
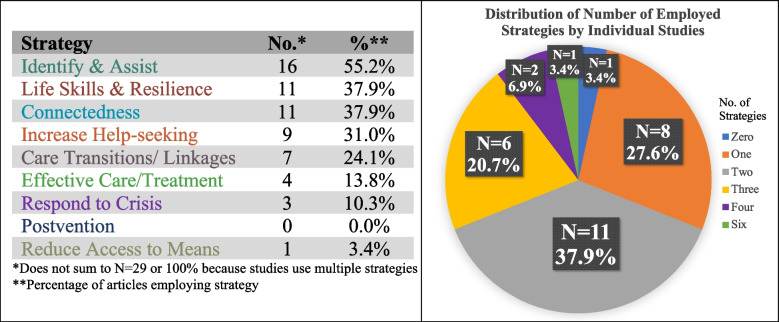
The number of suicide prevention resource center (SPRC) intervention strategies employed and level of comprehensiveness

Figure 3 shows the number of intervention strategies employed and the level of comprehensiveness of the studies, as defined by the SPRC (SPRC, 2020). Intervention strategies primarily focused on identifying and assisting persons at risk, appearing in 16 (55.2%) of the studies. These strategies consisted of training community members such as youth/peer leaders (Antonio et al., 2020; Chung-Do et al., 2015; Walker et al., 2009), adult gatekeepers (Story et al., 2016), mental health professionals (Capps et al., 2019; LoParo et al., 2019), extension agents (Robertson et al., 2021), teachers/school staff (Kohlbeck et al., 2021; Schmidt et al., 2015), and health providers (Christensen-LeCloux et al., 2021; LeCloux et al., 2020; McFaul et al., 2014). Other common strategies included enhancing life skills and resilience (n = 11, 37.9%), promoting supportive relationships and community connectedness (n = 11, 37.9%), and increasing help seeking behavior (n = 9, 31.0%).

Most interventions did not combine multiple target areas of the SPRC framework, with 65.5% using either a combination of two (n = 11) or single-based strategies (n = 8). One study that distributed video-enabled tablets to VA patients used no SPRC-level strategy (Gujral et al., 2022). While the intervention directly addressed a rural-specific access issue through reducing barriers for people who live far from mental healthcare services or who have time, transportation, or mobility restrictions, it did not meet criteria as laid out by the SPRC framework. Only one intervention employed six strategies, which used a combination of three interventions (a referral mechanism, mindfulness exercises, and a social marketing campaign) to promote behavioral health resiliency and decrease suicide risk factors among youth and young adults in rural Wisconsin (Kohlbeck et al., 2021). No study provided a postvention plan, and only one focused on reducing access to lethal means for suicide (Capps et al., 2019).

### Summary of Findings

Despite the wide variation in methodologies, trial types, and measured outcomes, some general themes were observed in the retrieved studies' main findings. All included papers built upon an increasing evidence-base for community-based suicide interventions. Overall, studies offered promising solutions to reduce suicide in rural areas, though higher methodological rigor is needed to better identify the most effective interventions. Only one article reported adverse impacts of the intervention, using qualitative themes to illustrate how a male-targeted campaign failed to surpass cultural and treatment barriers to increase access to and utilization of treatment (Ewert, 2021). Formative studies demonstrated appropriate reach and other measures of participation (Brancu et al., 2020; Capps et al., 2019; Chung-Do et al., 2015), as well as positive implementation outcomes, including fidelity, feasibility, and acceptability (Bailey et al., 2022; LeCloux et al., 2020; Trout et al., 2018; Wexler et al., 2017b). However, there was a lack of research measuring implementation cost, penetration, and sustainability. Among effectiveness studies, relatively few examined clinical outcomes, and none linked program impacts to reductions in completed suicides. However, the emerging evidence indicated general reductions in differential measures such as suicidal risk and ideation (Schmidt et al., 2015; Wyman et al., 2010), inpatient psychiatric admissions/referrals (Capps et al., 2019; Kim et al., 2022), and other related clinical outcomes such as stress (Kohlbeck et al., 2021), affect (Barnett et al., 2020), and substance use (Wexler et al., 2017a). Effectiveness papers also showed positive outcomes in participant understanding of intervention content (LoParo et al., 2019; McFaul et al., 2014; Robertson et al., 2021).

## Discussion

This scoping review aimed to comprehensively explore the key characteristics of the literature focusing on community-based suicide prevention initiatives for rural communities in the US. This study documents intervention characteristics as well as the diverse methodological approaches employed. Overall, the findings presented from the reviewed papers were largely positive, with studies demonstrating promising effects when evaluating process, implementation, and effectiveness outcomes. However, the lack of a comprehensive evidence-base framework limited the ability to fully evaluate the findings. Additionally, the inclusion of 29 articles suggests a lack of studies despite the consistently higher and growing rates of rural suicides as compared to urban areas (CDC, 2023). This dearth in research echoes existing gaps in the literature on overall suicide trends and considerations in rural US settings, with the majority of studies on suicidality focusing on urban populations (Handley et al., 2011). More focus on rural populations is needed for both development of community-based initiatives as well as for understanding contextual factors related to suicidal behavior so that targeted suicide preventions can be developed (Steelesmith et al., 2019).

Additionally, consistency is needed in accurately labeling and defining a region as “rural”. Rurality is a multidimensional concept (Coburn et al., 2007). The Alaskan Bush, remote Appalachia, and Midwestern farmlands contain vastly different cultural, infrastructure, climate, and geographic variations. Simply designating these environments as “rural” creates an oversimplified categorization, which generalizes disparate and unique landscapes (Casant & Helbich, 2022). A lack of conceptual clarity and cohesive definitions of the study context can make it challenging to have confidence in the conclusions formed by researchers and to build an organized and comprehensive evidence base (Coburn et al., 2007). At the very least, accurately labeling rural contexts through such approaches as Rural–Urban Commuting Area (RUCA) codes or the Office of Management and Budget (OMB) offers such clarity. With clearer definitions, researchers, policymakers, and rural practitioners can more appropriately identify, develop, and finetune strategies that better meet the mental health needs of various rural populations (Childs et al., 2022).

However, there are also limitations to existing rural taxonomies, which often do not incorporate important demographic, cultural, environmental, and economic differences. Highlighting and incorporating these differences can aid in identifying distinct mental healthcare issues and effective strategies for addressing those concerns (Hart et al., 2005). For example, although no included studies in Alaska provided official rural definitions, multiple offered such descriptions, giving rich details of rural characteristics specific to the Alaskan circumpolar context (Allen et al., 2009, 2018; Barnett et al., 2020; Mohatt et al., 2014; Trout et al., 2018; Wexler et al., 2017a, b). This included describing remote villages as not being connected by road systems but instead through seasonal small aircraft, boat or snowmobile, as well as being served by an under-resourced healthcare system with many non-Native service providers.

Multiple Alaskan studies also reported conceptual frameworks that guided intervention design, a finding not consistent with the majority of included studies. When tailoring community-based approaches, it is important to have an underlying framework relevant to the studied region and population context. Culturally-informed theoretical frameworks that explicitly explain and map known facts about suicide allow for guidance in developing interventions that reliably identify risks for future occurrences of suicidal behavior, thereby improving the effectiveness of suicide prevention (Chu et al., 2010, 2017). Multiple studies in the Alaskan cohort offer such examples which are based on Indigenous knowledge and decolonial approaches. These frameworks informed intervention design and data collection strategies, providing guideposts for context-specific measurements for mental health and wellbeing. These frameworks acknowledge historic traumas and underlying suicidal risks while also promoting collective strength and abilities.

While over a third of the included papers focused on Indigenous youth, a population disproportionately at great risk for suicide-related deaths and behaviors in the US (Mpofu et al., 2023), these studies were limited to Alaska and to a lesser extent Hawaii. More interventions are needed to serve other AI/AN communities throughout the US. In addition, gaps in the included evidence-base indicate that more intervention efforts are needed for other at-risk populations in the rural context, including sexual and gender minorities, young men, and agricultural workers.

More comprehensive approaches are also needed. Effective action towards reducing suicide at the community level requires multilevel strategies that match the rural context. This means approaches should require wraparound services such as effective mental health treatment, crisis management, and gatekeeper training to aid recognition of individuals potentially at risk. Additional strategies include creating organizational linkages for case management, reducing stigma through publicity and outreach campaigns, and promoting connectedness within marginalized and isolated communities. The SPRC (2020) offers a model to program developers for such an approach, illustrating multiple intervention strategies that comprise a comprehensive community-based suicide prevention approach. Future intervention development initiatives should consider integrating and linking various strategies to create a more inclusive and wide-ranging approach. More focus is also needed on providing protocols for postvention plans to respond to a suicide death effectively and compassionately. Additionally, efforts should address reducing access to lethal means. Lethal means safety efforts face challenges, especially considering the cultural and emotional attachments to firearms in rural populations (Pierre, 2019). However, action is needed, as access to guns is strongly related to elevated rural US suicide rates, far outpacing urban contexts in adults and adolescents (Buck-Atkinson et al., 2023; Mohatt et al., 2021; Pallin & Barnhorst, 2021; Runkle et al., 2023).

To ensure the development of contextually relevant and sustainable interventions, incorporating community-based participatory research (CBPR) methods is essential, as it can empower rural communities to actively shape suicide prevention efforts based on their unique needs, strengths, challenges, and cultural contexts (Kral & Kidd, 2018). CBPR emphasizes the active involvement of community members in all stages of research, and directly supports the call for comprehensive services and integrated strategies by engaging local stakeholders at all levels in the design, implementation, and evaluation of interventions (Parker et al., 2020; Scheyett et al., 2024). CBPR also supports the sustainability of interventions. When local stakeholders are actively involved in intervention development, they are more likely to continue and adapt the programs after external funding or support has ended (Hacker et al., 2012). CBPR approaches also help to build trust in communities, which can be particularly salient for programs implemented in rural areas (Burhansstipanov et al., 2005; Lister & Joudrey, 2023). Thus, by building local capacity, CBPR strengthens rural communities’ ability to respond to suicide-related issues independently and effectively.

Lastly, while quality appraisal is not conducted in scoping reviews, this review points to the need for more robust methodological approaches. All but one of the studies were noncontrolled investigations. Relatively few reported clinical outcomes, and none linked intervention impacts to reductions in completed suicides. While some studies reported differences in death by suicide pre- and post-intervention implementation, causal inferences could not be made (Capps et al., 2019; Kohlbeck et al., 2021). More indications on programming impacts on the numbers of completed or attempted suicides are needed. Additionally, research should examine differential effects, including psychiatric admissions and other relevant and underlying outcomes such as mood, affect, and substance use (Hofstra et al., 2020). Future research should also be aware of limitations of differential effects across specific characteristics of settings and populations. For example, psychiatric admissions can be very effective in identifying and preventing suicidal behaviors for some groups, while also exacerbating pervasive distress for others (Iudici et al., 2022). Furthermore, to address the methodological gaps highlighted in this review, we recommend incorporating longitudinal and mixed-methods research designs. Longitudinal studies would allow for tracking the impact of interventions over time, while mixed-methods approaches could provide a more holistic view by capturing both statistical trends and the lived experiences of rural communities. This would enhance our understanding of the long-term sustainability and effectiveness of rural suicide prevention programs.

### Limitations

Several limitations must be noted regarding this review. The search was limited to English-only articles and empirical papers, leaving out other grey literature and non-English studies. These results may therefore not be generalizable to an international perspective. Additionally, the exclusion of grey literature may have omitted valuable work conducted by rural providers that is not reported in academic journals. Furthermore, our search strategy relied on variations of “rural” as a key term, which may have inadvertently excluded studies specifically focused on farming and ranching communities. While this aligns with the study’s scope, it is worth noting that the USDA has funded significant research on suicide prevention in agricultural communities, and those studies may not be represented in this review. Lastly, this review did not assess the methodological rigor of the included studies. However, this relates to the purpose of scoping reviews, which is to not collate and assess empirical evidence but to review the current state of knowledge within the literature and illustrate gaps, thereby establishing and advocating for a new research agenda and providing policy recommendations for the delivery of future programs.

## Conclusion

The suicide rate in the rural US is nearly twice that of urban areas and has been steadily growing over the past two decades. The burden of suicide is great, costing enumerable emotional and psychological tolls to individuals, families, communities, and society. Community-based suicide prevention initiatives that are comprehensive in their approach have been called for to address the need for holistic strategies relevant to the varied landscapes, populations, and needs comprised within rural communities. This scoping review was systematically undertaken and highlights the dearth of literature on prevention programming in an often-overlooked context. It also offers investigators a baseline guide to the existing evidence on community-based approaches, outlining growing patterns and offering recommendations for future program development and implementation. As suicidal behavior is complex and determined by numerous intertwined factors, complexities arise in developing interventions that measure the heterogeneity in suicidal behavior and outcomes. Nonetheless, given the high priority of this public health issue, continued development and rigorous testing of rural community-based initiatives are needed.

## Supplementary Information

Below is the link to the electronic supplementary material.

Supplementary file1 (DOCX 20.5 KB)
