# Supplementary material for: Community-based Suicide Interventions in Rural United States: A Scoping Review
Source: Community Ment Health J. 2025 Jul 23;61(8):1437–60. doi: 10.1007/s10597-025-01480-x (PMC12647199; doi:10.1007/s10597-025-01480-x)
Supplement: Supplementary file 1 — Supplementary file1 (DOCX 20.5 KB) [file 10597_2025_1480_MOESM1_ESM.docx]

**Appendix A:** Full list of search terms and number of results from each database

**EBSCO Medline**
**Date Searched**: 8/16/2022

**Applied Database Supplied Limits**: Scholarly (Peer Reviewed) Journals; Date of Publication: 2000-; English Language; Journal & Citation Subset: MEDLINE, In Process, In Data Review

**Number of Results**: 900

**Full Search Strategy:**

( (MH "Suicide+") OR AB suicid* OR TI suicid* ) AND ( (MH "Crisis Intervention") OR (MH "Program Evaluation+") OR (MH "Pilot Projects") OR AB (intervention* OR program* OR initiativ* OR strateg* OR "crisis response" OR preventi* OR service* OR screening OR implement*) OR TI (intervention* OR program* OR initiativ* OR strateg* OR "crisis response" OR preventi* OR service* OR screening OR implement*) ) AND ( (MH "rural population") OR AB (rural OR frontier OR remote OR non-urban OR non-metro) OR TI (rural OR frontier OR remote OR non-urban OR non-metro) )

**Ebsco PsycINFO**

**Date Searched**: 8/16/2022

**Applied Database Supplied Limits**: Publication Year: 2000-; Peer Reviewed; English

**Search modes** - Boolean/Phrase

**Number of Results**: 680

**Full Search Strategy:**

Suicid* AND ( AB (intervention* OR program* OR initiativ* OR strateg* OR "crisis response" OR preventi* OR service* OR screening OR implement*) OR TI (intervention* OR program* OR initiativ* OR strateg* OR "crisis response" OR preventi* OR service* OR screening OR implement*) ) AND ( AB (rural OR frontier OR remote OR non-urban OR non-metro) OR TI (rural OR frontier OR remote OR non-urban OR non-metro) )

**Scopus**
**Date Searched:** 8/16/2022

**Applied Database Supplied Limits:** English, Pub Year 2000-2022, Journal Article

**Number of Results:** 1,384

**Full Search Strategy:**

## TITLE-ABS-KEY ( suicid* )  AND  TITLE-ABS-KEY ( intervention*  OR  initiativ*  OR  strateg*  OR  preventi*  OR  service*  OR  screening  OR  implement*  OR  "crisis response" )  AND  TITLE-ABS-KEY ( rural*  OR  frontier  OR  remote  OR  non-urban  OR  non-metro )  AND  ( LIMIT-TO ( PUBYEAR ,  2022 )  OR  LIMIT-TO ( PUBYEAR ,  2021 )  OR  LIMIT-TO ( PUBYEAR ,  2020 )  OR  LIMIT-TO ( PUBYEAR ,  2019 )  OR  LIMIT-TO ( PUBYEAR ,  2018 )  OR  LIMIT-TO ( PUBYEAR ,  2017 )  OR  LIMIT-TO ( PUBYEAR ,  2016 )  OR  LIMIT-TO ( PUBYEAR ,  2015 )  OR  LIMIT-TO ( PUBYEAR ,  2014 )  OR  LIMIT-TO ( PUBYEAR ,  2013 )  OR  LIMIT-TO ( PUBYEAR ,  2012 )  OR  LIMIT-TO ( PUBYEAR ,  2011 )  OR  LIMIT-TO ( PUBYEAR ,  2010 )  OR  LIMIT-TO ( PUBYEAR ,  2009 )  OR  LIMIT-TO ( PUBYEAR ,  2008 )  OR  LIMIT-TO ( PUBYEAR ,  2007 )  OR  LIMIT-TO ( PUBYEAR ,  2006 )  OR  LIMIT-TO ( PUBYEAR ,  2005 )  OR  LIMIT-TO ( PUBYEAR ,  2004 )  OR  LIMIT-TO ( PUBYEAR ,  2003 )  OR  LIMIT-TO ( PUBYEAR ,  2002 )  OR  LIMIT-TO ( PUBYEAR ,  2001 )  OR  LIMIT-TO ( PUBYEAR ,  2000 ) )  AND  ( LIMIT-TO ( DOCTYPE ,  "ar" ) )  AND  ( LIMIT-TO ( LANGUAGE ,  "English" ) )

**Cinahl**
**Date Searched**: 8/16/2022

**Applied Database Supplied Limits:** Published Date: 2000-; English Language; Peer Reviewed

**Number of Results:** 613

**Full Search Strategy:**

( (MH "Suicide+") OR AB suicid* OR TI suicid* ) ) AND ( (MH "Crisis Intervention") OR (MH "Program Evaluation+") OR (MH "Pilot Projects") OR AB (intervention* OR program* OR initiativ* OR strateg* OR "crisis response" OR preventi* OR service* OR screening OR implement*) OR TI (intervention* OR program* OR initiativ* OR strateg* OR "crisis response" OR preventi* OR service* OR screening OR implement*) ) AND ( (MH "rural population") OR AB (rural OR frontier OR remote OR non-urban OR non-metro) OR TI (rural OR frontier OR remote OR non-urban OR non-metro) )
